# Morphine-induced microglial immunosuppression via activation of insufficient mitophagy regulated by NLRX1

**DOI:** 10.1186/s12974-022-02453-7

**Published:** 2022-04-12

**Authors:** Jialing Peng, Jingrui Pan, Hongxuan Wang, Jingjing Mo, Lihuan Lan, Ying Peng

**Affiliations:** 1 Department of Neurology, Sun Yat-Sen Memorial Hospital, Sun Yat-Sen University, No. 107 West Yanjiang Road, Guangzhou, 510120 China; 2 Guangdong Provincial Key Laboratory of Malignant Tumor Epigenetics and Gene Regulation, Sun Yat-Sen Memorial Hospital, Sun Yat-Sen University, Guangzhou, China

**Keywords:** NLRX1, Morphine, Microglia, Mitophagy, Immunosuppression

## Abstract

**Background:**

Chronic morphine exposure induces immunosuppression in the peripheral and central nervous system, resulting in susceptibility of patients to invading pathogens. Mitophagy is a crucial regulator of inflammation, and dysregulated mitophagy may cause immunosuppression, but whether mitophagy is linked with morphine-induced immunosuppression in the brain remains unknown. NLRX1 is the only mitochondrially localized NOD family receptor protein which serves as a critical regulator in immunity and mitophagy activation, but it remains an enigma how NLRX1 functions in the crosstalk between microglial inflammatory defense and mitophagy in the presence of morphine.

**Methods:**

Primary microglia and astrocytes, BV2 and MA cell lines were utilized. Mice were stimulated with repeated morphine treatment to mimic chronic morphine exposure, and activation of mitophagy, lysosomal functions, and inflammation were assayed in specific brain regions and immune organs with or without NLRX1-silencing.

**Results:**

Morphine induced microglial mitophagy in a LC3 (microtubule-associated proteins light chain 3)-dependent manner, which was mediated by NLRX1. Contrastingly, morphine impaired lysosomal functions, including generation, acidification and mitophagosome–lysosome fusion, thus leading to insufficient mitophagy activation in microglia. NLRX1-silencing inhibited mitophagy activity and rescued lysosomal functions including generation and acidification in microglia. The NLRX1-mediated incomplete mitophagy in microglial cells contributed to immunosuppression and vulnerability towards pathogenic challenge after morphine treatment. In vivo, NLRX1-mediated microglial mitophagy activation by morphine was mainly located in the murine brain cortex, striatum, and cerebellum, where NLRX1 functioned as a negative immune regulator and facilitated septic shock. Collectively, microglial immune responses to septic shock were amenable to NLRX1 silencing in the brain with morphine treatment.

**Conclusion:**

Morphine activated insufficient mitophagy in microglia which was regulated by NLRX1, ultimately leading to host immunosuppression and susceptible conditions in the brain.

**Supplementary Information:**

The online version contains supplementary material available at 10.1186/s12974-022-02453-7.

## Introduction

Morphine is widely used to treat chronic pain clinically, including cancer-related and non-cancer-related pain [1]. However, chronic morphine treatment will lead to deleterious host innate immunity impairment [2], which potentiates pathogenic infections such as HIV. Immunosuppressive complications will limit the clinical efficacy of morphine. However, the exact mechanisms involved in morphine-induced immunosuppression, especially in the brain, still remain not fully clarified.

Mitochondria have emerged as critical mediators in the regulation of inflammation and host innate immunity [3]. Mitochondrion sustains constant fission and fusion to maintain physiological functions and cellular homeostasis. Mitophagy, a selective form of autophagy, eliminates damaged or excess mitochondria in response to cellular stress. During mitophagy, mitophagosomes, the double-membraned vesicles, engulf and encapsulate damaged mitochondria and are shuttled to lysosomes for further proteolytic degradation [4]. Multiple signaling mediators in innate immunity participate in mitophagy regulation, underlying the crosstalk between mitophagy and host inflammatory response [5]. Dysregulated mitophagy may orchestrate exuberant inflammasome activation or immunosuppression and various pathogens would be able to manipulate mitophagy facilitating pathogenesis during infection [6]. Generally, mitophagy is initiated through two major pathways, including the PINK1–Parkin axis and mitophagy receptors which contain an LC3 interacting region (LIR) motif for LC3-decorated autophagosome recruitment [7, 8]. Classically, the PINK1-Parkin pathway depended on the mitochondrial membrane potential (*Δψm*) and functions in an ubiquitin-dependent pattern [9]. On the other hand, several mitophagy receptors have been identified, among these receptors, NOD-like receptor X1 (NLRX1) is the only mitochondrially localized NOD family receptor protein, which was recently reported to function as a novel mitophagy receptor during *L. monocytogenes* infection to evade host killing [10]. NLRX1 contains a LIR motif for binding with LC3, a prerequisite for mitophagy receptor. However, the proposed mechanisms by which NLRX1 mediates mitophagy remain unknown and require further elucidation. So far, NLRX1 reportedly appears to be a versatile anti-inflammatory regulator during various pathogenic microorganism infection. NLRX1 mediated MAVS signaling and inhibited production of IFNs to facilitate the survival of viruses during mitochondrial antiviral immunity [11]. Contrastingly, NLRX1-deficient mice tended to be susceptible to LPS-induced septic shock, which resulted from aberrant immune responses [12]. In summary, NLRX1 seemed to serve as a critical negative regulator in immunity. Besides, intriguing data also revealed that NLRX1 manipulated virus-induced autophagy for host defense [13]. Taken together, NLRX1-mediated mitophagy may play an important role in pathogen–host cell interactions.

Microglial cells, which serve as the resident phagocytic and immune cells in the brain, have been reported to be pivotal mediators in infectious neuroinflammation in patients receiving morphine treatment [14]. Aberrant expression of immune-related genes (such as *IL-1β*, *IL-6*, *IL-18*, *TNF-α*, and so on) in microglia might inhibit microglial activity and endanger the central nervous system [15]. Severe or even fatal bacterial sepsis occurs frequently once invading pathogens attack, which might account for the high susceptibility to bacterial infection induced by morphine [16]. In the present study, we aimed to clarify the role of microglial mitophagy in the induction of cerebral immunosuppression and its regulative mechanisms after morphine treatment, as well as the selective brain regions [17] where microglial mitophagy mainly served as the immunoregulatory element in morphine-treated mice.

## Materials and methods

### Reagents

Morphine was provided by Sun Yat-sen Memorial Hospital, which was approved by Guangdong Medical Products Administration. CCCP (10 μM, HY-100941), Mdivi-1 (20 μM, HY-15886) and Bafilomycin A1 (20 nM, HY-100558) were purchased from MedChemExpress. Torin1 (250 μM, SC0245) was purchased from Beyotime. Puromycin (Sigma, P7255) was used to select stably expressed cells.

### Cell culture and RNA interference

Primary microglial cells were isolated and cultured according to our previous protocol [18] and the purity was verified as 95% or higher by immunofluorescence staining with Iba-1 (Additional file 1: Fig. S1A). For primary astrocytes, the cells were cultured in DMEM (HyClone, SH30243.01) containing 10% fetal bovine serum (FBS) (Gibco, 10099–141) and 1% penicillin/streptomycin. After the cells reached confluence (about 7 days), the cells were subcultured. After 30-min pre-adherence, the medium containing nonadherent cells was replaced. Subculture was performed three times every 4 days as suggested [19]. The purity of astrocyte was confirmed by fluorescence staining with GFAP (Additional file 1: Fig. S1B). BV2 cells and MA cells were purchased from American Type Culture Collection (ATCC). BV2 cells were cultured in DMEM/F12 medium (Gibco, 11330-032) while MA cells in DMEM high glucose. Cells were maintained in complete medium containing 10% FBS and 1% penicillin/streptomycin in a 5% CO_2_ incubator at 37 °C. For siRNA transfection, the NC-siRNA and NLRX1-siRNA were purchased from RiboBio Co., Ltd (Guangzhou, China). Transfection was performed using Lipofectamine 3000 reagents (Invitrogen, L3000001) and the sequence targeting NLRX1 was as follows: sense, 5′-GCCACAGAAGCUAUCCAAAdTdT-3′, anti-sense 5′-CGGUGUCUUCGAUAGGUUUdTdT-3′.

### DNA, RNA isolation and real-time quantitative PCR (qPCR)

Genomic DNA was isolated using TIANamp Genomic DNA kit (TIANGEN, DP304-03). *mtDNA* (mitochondrial DNA, the conserved sequence in the D-loop region) copy number was normalized to nuclear DNA (*Hbb*, β-globin) gene. Total RNA was isolated by TRIzol reagent (Takara, #9109) and then transcribed to cDNA using PrimeScript RT Reagent Kit (Takara, #RR037A). The qPCR was performed in Applied Biosystems QuantStudio 5 (ThermoFisher). The following primers were used:

(Mouse) *mt-DNA* forward, GCCCATGACCAACATAACTG; (Mouse) *mt-DNA* reverse, CCTTGACGGCTATGTTGATG; (Mouse) *Hbb* (β-globin) forward, AGGCAGAGGCAGGCAGAT; (Mouse) *Hbb* (β-globin) reverse, GGCGGGAGGTTTGAGACA; (Mouse) *IL-1β* forward, TGCCACCTTTTGACAGTGATG; (Mouse) *IL-1β* reverse, AAGGTCCACGGGAAAGACAC, (Mouse) *IL-6* forward, AGGATACCACTCCCAACAGACCT; (Mouse) *IL-6* reverse, CAAGTGCATCATCGTTGTTCATAC, (Mouse) *IL-18* forward, ATGCTTTCTGGACTCCTGCC; (Mouse) *IL-18* reverse, ATTGTTCCTGGGCCAAGAGG, (Mouse) *TNF-α* forward, ATGCTTTCTGGACTCCTGCC; (Mouse) *TNF-α* reverse, ATTGTTCCTGGGCCAAGAGG, (Mouse) *iNOS* forward, CTTGCCACGGACGAGAC; (Mouse) *iNOS* reverse, TCATTGTACTCTGAGGGCTGA, (Mouse) *NLRX1* forward, ACCTCACCGAGTGGTTTAGC; (Mouse) *NLRX1* reverse, TCACGGGGTCAACATGAACTG, (Mouse) *GAPDH* forward, TGACCTCAACTACATGGTCTACA; (Mouse) *GAPDH* reverse, CTTCCCATTCTCGGCCTTG, (Mouse) *Atp6v0d1* forward, CGCCACATGAGAAACCATGC; (Mouse) *Atp6v0d1* reverse, CTCAAAGCTGCCTAGCGGAT, (Mouse) *Atp6v0d2* forward, CTGGTTCGAGGATGCAAAGC; (Mouse) *Atp6v0d2* reverse, TCCAAGGTCTCACACTGCAC, (Mouse) *LAMP1* forward, CCAGAGCGTTCAACATCAGC; (Mouse) *LAMP1* reverse, ACAGGCTAGAGCTGGCATTC, (Mouse) *LAPTM4A* forward, TGCGTTCTTTTTGCCGTCTC; (Mouse) *LAPTM4A* reverse, GAATCAGCCAGCCCACTTGA.

### Co-immunoprecipitation and western blotting

Cells were washed in PBS and lysated in IP lysis buffer (Beyotime, P0027). 500 ug proteins were subjected to immunoprecipitation. 1 ug rabbit anti-NLRX1 antibody (Cell Signaling Technology, 13829 s) was added to lysate for 1 h, followed by 20 μl protein A agarose beads (Santa-Cruz, sc-2003) overnight. The beads were washed by cold PBS and eluted by boiling in 2 × loading buffer. The input and eluted fractions were then subjected to immunoblot analysis. Homophytic IgG was employed as a negative control. NLRX1 was used for equalization for IP.

For western blotting, cells or brains were homogenized in lysis buffer (Beyotime, P0013) with complement of phenylmethylsulfonyl fluoride (Beyotime, ST506). Equal amounts of protein lysates were used for immunoblot analysis. Following primary antibodies were used: HSP60 (Affinity, AF0184), Tim23 (Affinity, DF12052), LC3A/B (Cell Signaling Technology, 4108S), GAPDH (Cell Signaling Technology, 2118S), mTOR (Affinity, AF6308), p-mTOR (Proteintech, 67778-1). Image J was utilized to analyze the densitometry of bands and GAPDH was use as a loading control.

### IF/ICC immunofluorescence staining

After cardiac perfusion, brains of mice were removed and fixed in 4% paraformaldehyde solution at 4 °C, following by gradient dehydration in sucrose. Brains were then cut into thick sections (10 µm) by LEICA CM1950. The following primary antibodies were used: NLRX1 Rabbit antibody (Affinity, DF12124), MAP1LC3B Mouse antibody (ABclonal, A17424), Iba1 Goat antibody (Abcam, ab48004), GFAP Mouse antibody (Huabio, EM140707), CD31 Mouse antibody (Abcam, ab222783), NEUN Mouse antibody (Abcam, ab104224). The following secondary antibodies were used: Alexa Fluor 488 AffiniPure Donkey anti-Rabbit IgG (H + L) (Yeasen, 34206ES60, 1:200), Alexa Fluor 647 AffiniPure Donkey Anti-Mouse IgG (H + L) (Yeasen, 34113ES60, 1:100), Cy3-labeled Donkey Anti-Goat IgG(H + L) (Beyotime, A0502, 1:250), Alexa Fluor 555-labeled Donkey Anti-Mouse IgG(H + L) (Beyotime, A0460, 1:500). The images were acquired and analyzed by a confocal microscope (LSM 880 with Airyscan). To elucidate the cellular location of NLRX1 and LC3B in *vivo*, 11–12 randomly selected fields per group (*n* = 3 mice) were used to calculate the ratio of NLRX1 + cells in microglia, NLRX1 + cells in LC3B + microglia and the ratio of microglia in NLRX1 + cells.

For ICC, cells were cultured in glass bottom cell cultured dishes (NEST, 801,001) and washed by cold PBS. After fixation by 4% paraformaldehyde solution, permeabilizationwas performed in 0.3% Triton™ X-100 in PBS. After blocking for 1 h, the cells were incubated by primary antibodies as follows: NLRX1 Rabbit antibody (Affinity, DF12124), MAP1LC3B Mouse antibody (ABclonal, A17424), LAMP1 Rabbit antibody (Bioss, bs-1970R), Iba1 Goat antibody (Abcam, ab48004), GFAP Mouse antibody (Huabio, EM140707), Tim23 Rabbit antibody (Affinity, DF12052), HSP60 Rabbit antibody (Affinity, AF0184), TFEB Rabbit antibody (Affinity, AF7015). The secondary antibodies were Alexa Fluor 555-labeled Donkey Anti-Rabbit IgG(H + L) (Beyotime, A0453, 1:500) and Alexa Fluor 488-labeled Goat Anti-Mouse IgG(H + L) (Beyotime, A0428, 1:500), Cy3-labeled Donkey Anti-Goat IgG(H + L) (Beyotime, A0502, 1:250), Alexa Fluor 555-labeled Donkey Anti-Mouse IgG(H + L) (Beyotime, A0460, 1:500). Immunofluorescence images were captured by confocal laser scanning microscope (FV10i, Olympus). The co-localization was analyzed by Image-Pro Plus 6.0 (Media Cybernetics, USA). For the co-localization analysis of NLRX1 and LC3B, Pearson’s correlation coefficient of the single cell from at least 6 randomly selected fields in 3 independent experiments per group were analyzed. For the analysis of TFEB nuclear translocation, 25 randomly selected fields in 3 independent experiments per group were analyzed. For the co-localization analysis of LAMP1 + lysosomes and LC3B + particles, approximately, 25–40 cells from at least 5 randomly selected fields in 3 independent experiments per group were analyzed. The overlap coefficient, lysosomal phagocytosis ratio of LC3B and LC3 + phagosomes per cell were analyzed and plotted.

### Adenovirus mCherry‐GFP‐LC3B transfection

BV2 cells in 12-well plates were infected by 40 MOI (multiplicity of infection) adenovirus expressing mCherry‐GFP‐LC3B fusion protein (Beyotime, C3011) and the LC3B-positive autophagosomes were detected by a confocal microscope (LSM 980 with Airyscan). mCherry (red)- and GFP (green)-positive dots meant the aggregation of LC3B-positive autophagosomes. When the autophagosomes were infused by lysosomes, the GFP protein quenched in the acidic environment and mCherry protein was stable. More than 50 cells per group from 3 independent experiments were analyzed.

### Electron microscopy assays

Cells were divided into four groups: control group; morphine group; morphine + NC-siRNA group; morphine + NLRX1-siRNA group. Cells were quickly collected and fixed in 2.5% glutaraldehyde at 4 °C overnight. After washing by 0.1 M PBS for three times, samples were fixed in 1% osmic acid and then dehydrated in a series of graded ethanol. The cells were then embedded and 70-nm ultra-thin sections were stained by 3% uranyl acetate–lead citrate. The images were captured by an electron microscope (HITACHI 7800).

### JC-1 (5,5′,6,6′-tetrachloro-1,1′,3,3′-tetraethyl-benzimidazolyl carbocyanine iodide)

To measure the mitochondrial membrane potential (*Δψm*), JC-1 (Beyotime, C2006) assay was performed according to manufacturers’ instructions. After incubation with 10 μg/mL JC-1 at 37 °C for 20 min, cells were washed and detected by a flow cytometer (LSR II, BD). The *Δψm* was revealed by the ratio of red intensity (JC-1 aggregates) to green intensity (JC-1 monomers).

### Mitochondrial and lysosomal fluorescent probes and image quantitation

Cells were cultured in glass cultured dishes and incubated with LysoTracker Red DND-99 (Yeasen, 40739ES50) and MitoTracker® Green FM (Yeasen, 40742ES50) according to manufacturers’ instructions for 30 min. Gently replaced the medium with complete medium and captured images immediately by microscope (FV10i, Olympus). The morphologies of mitochondria and lysosomes were subsequently analyzed by Image J. Average size and circularity value of mitochondria were analyzed using a Particle Analysis in Image J from more than 25 cells in 3 independent experiments per group. Form factors of mitochondria were calculated by the reciprocal of circularity value according to a previous research [19]. The diameters of individual lysosomes (circular or oval shaped) were calculated and plotted as size distribution. As [19] suggested, lysosomes with diameter between 0.2 and 1 μm were recognized as normal lysosomes while those larger then 1 μm were considered as abnormal or vacuolar lysosomes.

LysoSensor™ Green DND-189 (Yeasen, 40767ES50) was utilized to detect the acidity of lysosomes. Cells were incubated with medium containing 1 uM probe for 30 min at 37 °C. Subsequently, the cells were suspended and detected by flow cytometer (LSR II, BD). The higher intensity indicated the enhanced lysosomal acidity.

### Cell viability assays

The Cell Counting Kit-8 (CCK8) assay (DOJINDO, CK04) was performed as manufacturers’ instructions. In brief, cells were pre-treated with morphine or vehicle and subsequent LPS treatment for 6 h, 12 h or 24 h. After washing by PBS, fresh medium containing 10% CCK8 solution was added to cells and incubated for 2 h at 37 °C. The absorbance values were read by a microplate reader (BMG POLARstar Omega) at 450 nm. The cell viability was revealed by the percentage of the optical density value in control group.

### Lentiviral silencing of NLRX1

Four shRNAs targeting NLRX1 and scrambled (Scr) shRNA plasmids were purchased from GeneCopoeia and the target sequences were as follows: scrambled, ACAGAAGCGATTGTTGATC; a, GCTGGACCGAAACAAACAACT; b, CCAGAAAGATCCCTTTAATTC; c, GCATCTATACCAGCTTTCTAC; d, GCTGCGCAAATACATGCTTCC. To generate shRNA-expressing lentivirus, 293Ft cells were transfected with 20/3 ug shRNA plasmids, packaging plasmids including 5 ug psPAX2 and 8/3 ug pMD2.G. After 72 h, the supernatant was collected and filtered by a 0.45-μm cell strainer. After concentration (Yeasen, 41101ES50), the biological titer of lentivirus were calculated and the optimal sequence was chosen according to the qPCR analysis of primary microglia (Additional file 1: Fig. S1D).

### Experimental animals

All experiments were under protocols approved by the Institutional Animal Care and Use Committee, Sun Yat-Sen University (Approval number, SYSU-IACUC-2018-000182). 6 to 8-week-old, male C57BL/6 mice were house-caged with a 12:12-h light–dark cycle and free to food and water. We made all efforts to minimize pain and suffering of the mice. The mice were randomly divided into groups as follows: (1) Control group; (2) Morphine group (15 mg/kg/day, subcutaneously, 7 d); (3) Lentivirus (Lv)-GFP (green fluorescence protein)-Scr-shRNA (bilaterally, intracerebroventricularly) + Morphine; (4) Lv-GFP-NLRX1-shRNA (bilaterally, intracerebroventricularly) + Morphine; (5) Lv-GFP-Scr-shRNA + morphine + LPS (intraperitoneally, 1 mg/kg, L2880, sigma); (6) Lv-GFP-NLRX1-shRNA + morphine + LPS. The mice were received LPS or saline at the last day and sacrificed at 6 h. The liver, spleen and thymus were weighed and the organ indexes were calculated as follows: organ index = (organ weight (mg)/bodyweight (10 g)) × 100%.

### Statistical analysis

The experimental values were expressed by mean ± SEM and the data were obtained from minimum of three repeats independently. Normal distribution test and Levene's test were performed. Student's t-test or Mann–Whitney U test by GraphPad Prism 8 software were employed to indicate statistical significance between two groups. One-way ANOVA or Kruskal–Wallis test were employed to indicate statistical significance among multiple groups. Values at *p* < 0.05 were considered statistically significant.

## Results

### Morphine induced NLRX1-mediated mitophagy in microglia

**Fig. 1:**
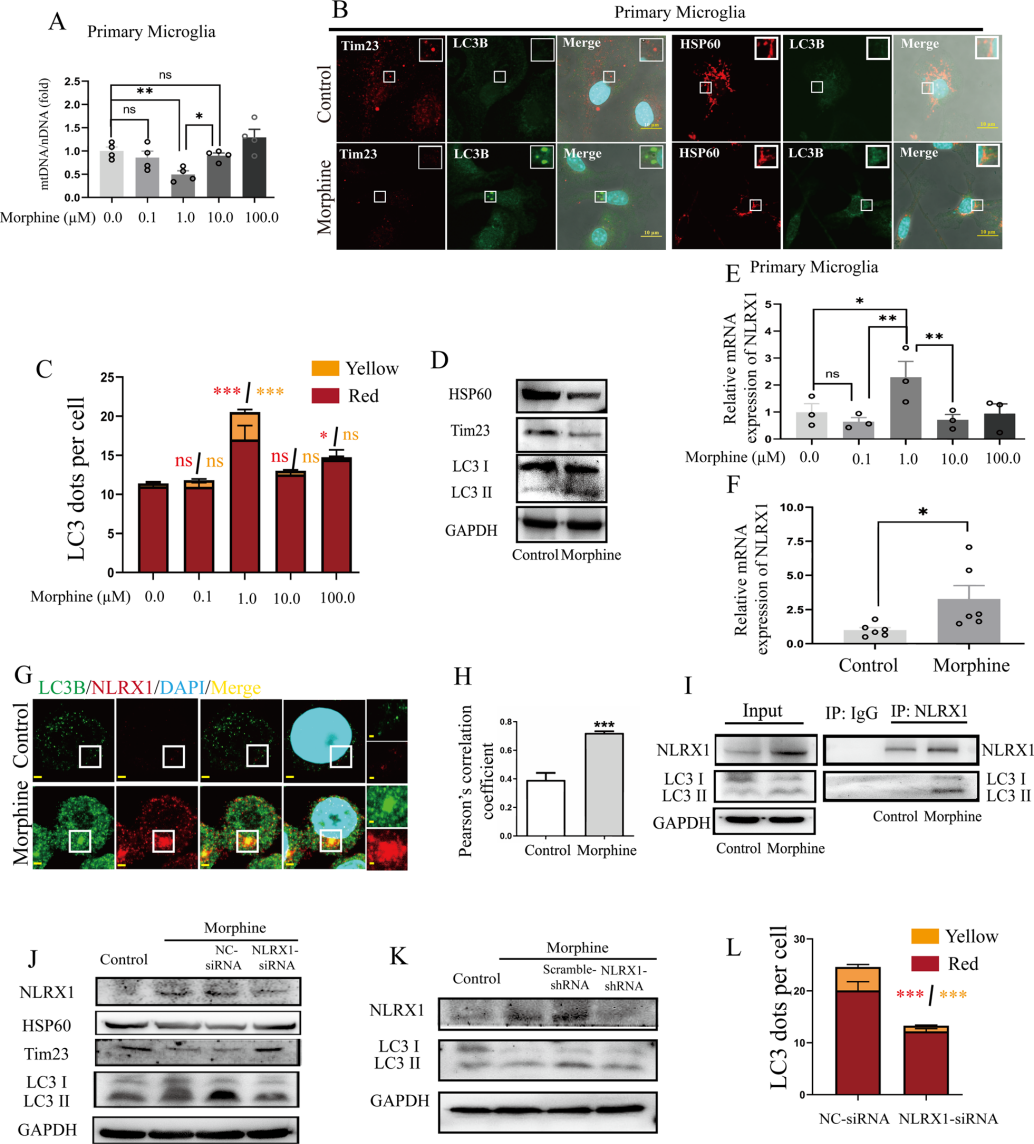
Morphine induced NLRX1-mediated mitophagy in microglia. **A** The mitochondrial DNA (*mtDNA*) levels were decreased significantly in primary microglia with 1.0 μM morphine as measured by mtDNA/nDNA analysis (*n* = 4). **B** Co-staining of HSP60 or Tim23 and LC3B in primary microglia. Bar = 10 μm. **C** The quantified results of AD-mCherry-GFP-LC3B transfection in BV2 cells with morphine treatment (*n* > 50 cells). The Kruskal–Wallis test was employed to indicate statistical significance. The results of mCherry-dots are shown in red and the mCherry-GFP-dots are shown in yellow. **D** Representative Western blots of BV2 cells in control or morphine treatment group (*n* = 3–6). **E** The expression of *NLRX1* mRNA was peaked in primary microglia with 1.0 μM morphine as measured by qPCR (*n* = 3). One-way ANOVA were employed in **A**, **E**. **F** Quantitative graphs of *NLRX1* mRNA in BV2 cells (*n* = 6). **G**, **H** Confocal microscopy analysis of NLRX1 (red), LC3B (green) and DAPI (light blue). Bar = 2 μm. The Pearson’s correlation coefficients of NLRX1 and LC3B were elevated in morphine-treated BV2 cells (**H**, *n* = 6–9 fields). **I** Co-immunoprecipitation analysis of NLRX1 and LC3 in BV2 cells (*n* = 3). **J**,** K** Western blots analysis of NLRX1-mediated mitophagy in BV2 cells by siRNA (**J**, *n* = 5) or shRNA (**K**, *n* = 3). **L** The quantified results of AD-mCherry-GFP-LC3B transfection in BV2 cells with NC-siRNA or NLRX1-siRNA pre-treatment (*n* > 50 cells). Data represent the mean ± SEM. Student's t-test or Mann–Whitney U test were used to measure significance between two groups. (* *p* < 0.05, ** *p* < 0.01, *** *p* < 0.001 and ns *p* > 0.05)

Primary microglia was treated with various concentrations of morphine (0.0, 0.1, 1.0, 10.0, 100.0 μM) for 24 h. Significant decrease of *mtDNA* copies were only observed in microglia treated with 1.0 μM morphine (Fig. 1A). Mitophagy could manipulate the *mtDNA* copies and we wondered whether mitophagy occurred in morphine-treated primary microglia. Thus, the LC3B puncta, which is an indication of mitophagosome formation, cooperating with a significant reduction of mitochondrial proteins (HSP60 and Tim23) were observed in primary microglial cells (Fig. 1B). To further confirm morphine regulated the mitophagosomes in a dose-dependent manner, BV2 cells were transduced with mCherry-GFP-LC3-expressing adenovirus (Fig. 1C). Significantly, accumulated mCherry-dots were observed in BV2 cells with 1.0 μM morphine treatment, indicating the aggregation of LC3B-decorated autophagosomes. In addition, the colocalized mCherry-GFP punctas increased in 1.0 μM morphine group, which suggested the possible defect of lysosomal function. More importantly, the mitochondrial protein levels of HSP60 and Tim23 were both decreased in morphine-treated BV2 cells (Fig. 1D, densitometric quantification data in Additional file 2: Fig. S2A). LC3 II, the lipidated form of LC3, played a critical role in mitophagosome membrane expansion and formation [18]. The conversion of LC3 I to LC3 II, as measured by the increased ratio of LC3 II/LC3 I, represented the maturation of phagosomes and activation of mitophagy. With morphine treatment, the conversion of LC3 I to LC3 II increased significantly (Fig. 1D, densitometric quantification data in Additional file 2: Fig. S2A). Taken together, 1.0 μM morphine exposure for 24 h might trigger microglial mitophagy.

Intriguingly, the mRNA expression of *NLRX1* peaked in microglial cells with 1.0 μM morphine treatment (Fig. 1E), underlying the possible associations between mitophagy and NLRX1. Then, BV2 cells were used and the *NLRX1* mRNA levels were similarly elevated after morphine treatment (Fig. 1F). NLRX1 was clarified to be a novel mitophagy receptor with a LIR motif and mediated mitophagy in an LC3-dependent manner [10]. Correspondingly, the LC3B punctas as well as NLRX1 upregulation were observed in the morphine-treated group (Fig. 1G). In addition, the Pearson’s correlation coefficient was increased in morphine group (Fig. 1H). Co-IP was then performed and LC3B was detected in the pull-downs by NLRX1 in morphine-treated BV2 cells, which proved the binding of NLRX1 to LC3B (Fig. 1I).

### NLRX1 was required for morphine-induced microglial mitophagy, but not astrocytes

To further elucidate the role of NLRX1, BV2 cells were pre-treated with NC-siRNA or NLRX1-siRNA. As expected, RNAi-mediated knockdown of NLRX1 increased the protein levels of HSP60 and Tim23 in morphine-treated BV2 cells (Fig. 1J, densitometric quantification data in Additional file 2: Fig. S2B). Additionally, the conversion of LC3 I to LC3 II was reversed, which indicated predominant role of NLRX1 in mitophagy. Also, BV2 cells stably expressing short hairpin RNAs (shRNAs) targeting NLRX1 or scramble-shRNA were generated. Similarly, the increase of NLRX1 and conversion of LC3 I to LC3 II induced by morphine were inhibited by NLRX1-shRNA (Fig. 1K, densitometric quantification data in Fig. Additional file 2: S2C). In addition, the cellular generation and accumulation of LC3B-phagosomes were also reversed by NLRX1-siRNA (Fig. 1L).

**Fig. 2:**
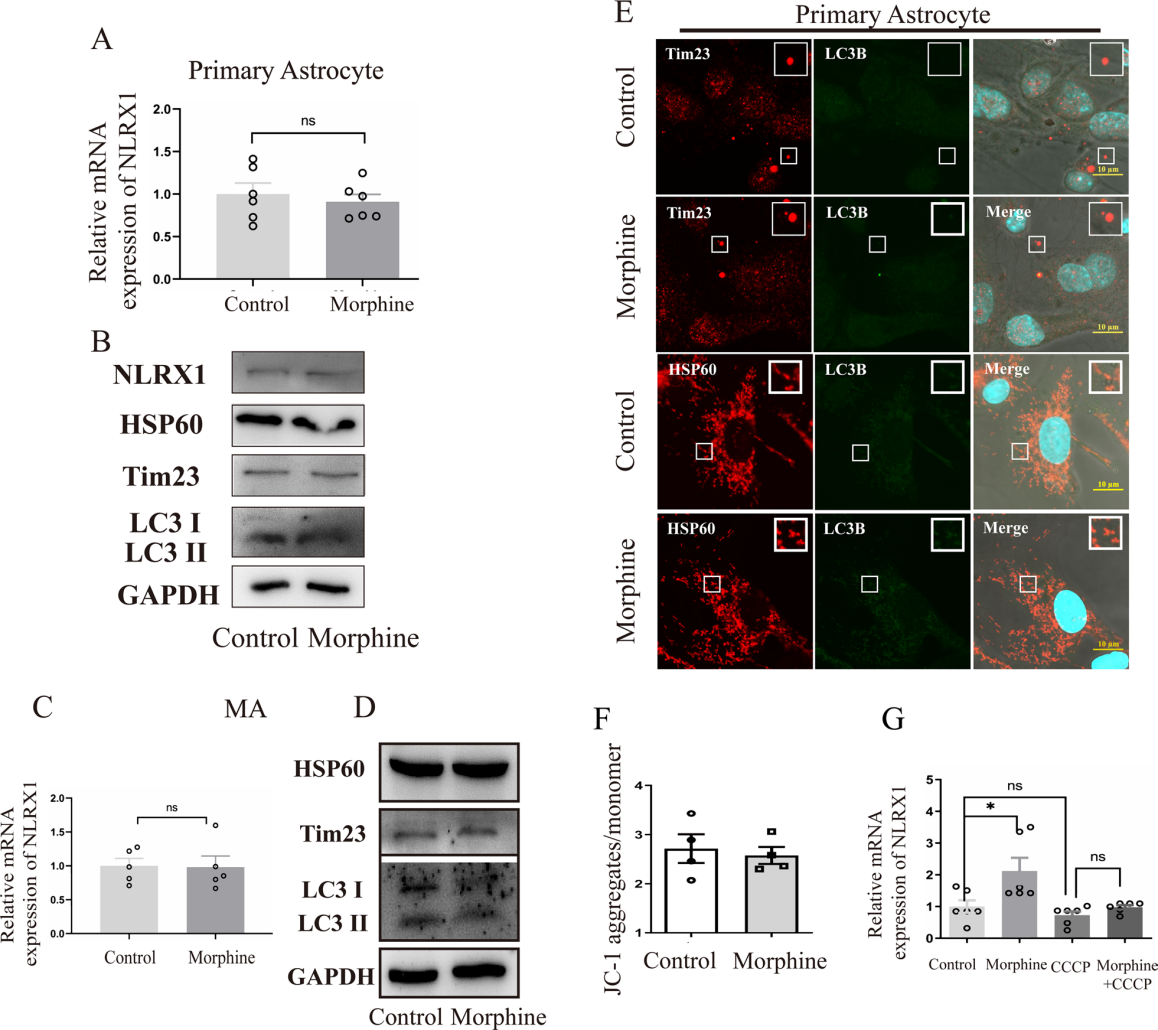
NLRX1-mediated mitophagy occurred in microglia independent of PINK1–Parkin pathway, but not astrocyte (**A**, **B**) Quantitative graphs of *NLRX1* mRNA (**A**, *n* = 6) and representative Western blots of mitophagy (**B**, *n* = 3) in primary astrocyte. Quantitative graphs of *NLRX1* mRNA (**C**, *n* = 6) and representative Western blots of mitophagy (**D**, *n* = 3) in MA cells. **E** Co-staining of HSP60 or Tim23 and LC3B in primary astrocyte. Bar = 10 μm. **F** The quantitative graphs of JC-1 assays as measured by flow cytometry (*n* = 4). **G** The *NLRX1* mRNA expression of BV2 cells with or without CCCP treatment (*n* = 6). Data represent the mean ± SEM. Two-sided Student’s t tests were used to measure significance between two groups (* *p* < 0.05, ** *p* < 0.01, *** *p* < 0.001 and ns *p* > 0.05)

However, we did not observe a significant increase of *NLRX1* mRNA in primary astrocytes and MA cells (Fig. 2A, C), a mouse astrocytic cell line, after morphine treatment. Consistently, the protein levels of HSP60 and Tim23 as well as conversion of LC3 I to LC3 II remained constant in astrocytes between the control and morphine group (Fig. 2B, D, densitometric quantification data in Additional file 1: Fig. S1C, Additional file 2: Fig. S2D). We further performed co-staining of HSP60 or Tim23 and LC3B in primary astrocytes, and no significant difference was observed between the control and morphine group (Fig. 2E). These findings collectively indicated that NLRX1 mediated mitophagy in morphine-treated microglia but not astrocyte.

### Morphine induced microglial mitophagy independent of PINK1–Parkin pathway

As the PINK1–Parkin pathway was involved in mitophagy, we further performed JC-1 assay and checked the *Δψm* in microglial cells, which was a critical precursor for PINK1–Parkin-dependent mitophagy. Virtually, we did not observe significant depolarization of mitochondria in cells after morphine treatment (Fig. 2F). Then, carbonyl cyanide *m*-chlorophenylhydrazone (CCCP), a critical mitophagy trigger, was added to induce significant loss of mitochondrial membrane potential and activation of the PINK1–Parkin pathway. The *NLRX1* mRNA expression remained unchanged after CCCP treatment (Fig. 2G), which suggested that depolarization of mitochondria was not an effective trigger in NLRX1-mediated mitophagy. Abnormal oligomerization in the mitochondria might be critical for the activation of NLRX1 and NLRX1-mediated mitophagy [10]. Herein, CCCP co-treated with morphine impeded the upregulation of NLRX1 induced by morphine, probably owing to the excessive elimination of mitochondria by CCCP-induced mitophagy.

### Morphine induced NLRX1-mediated dysfunctional mitophagy

**Fig. 3:**
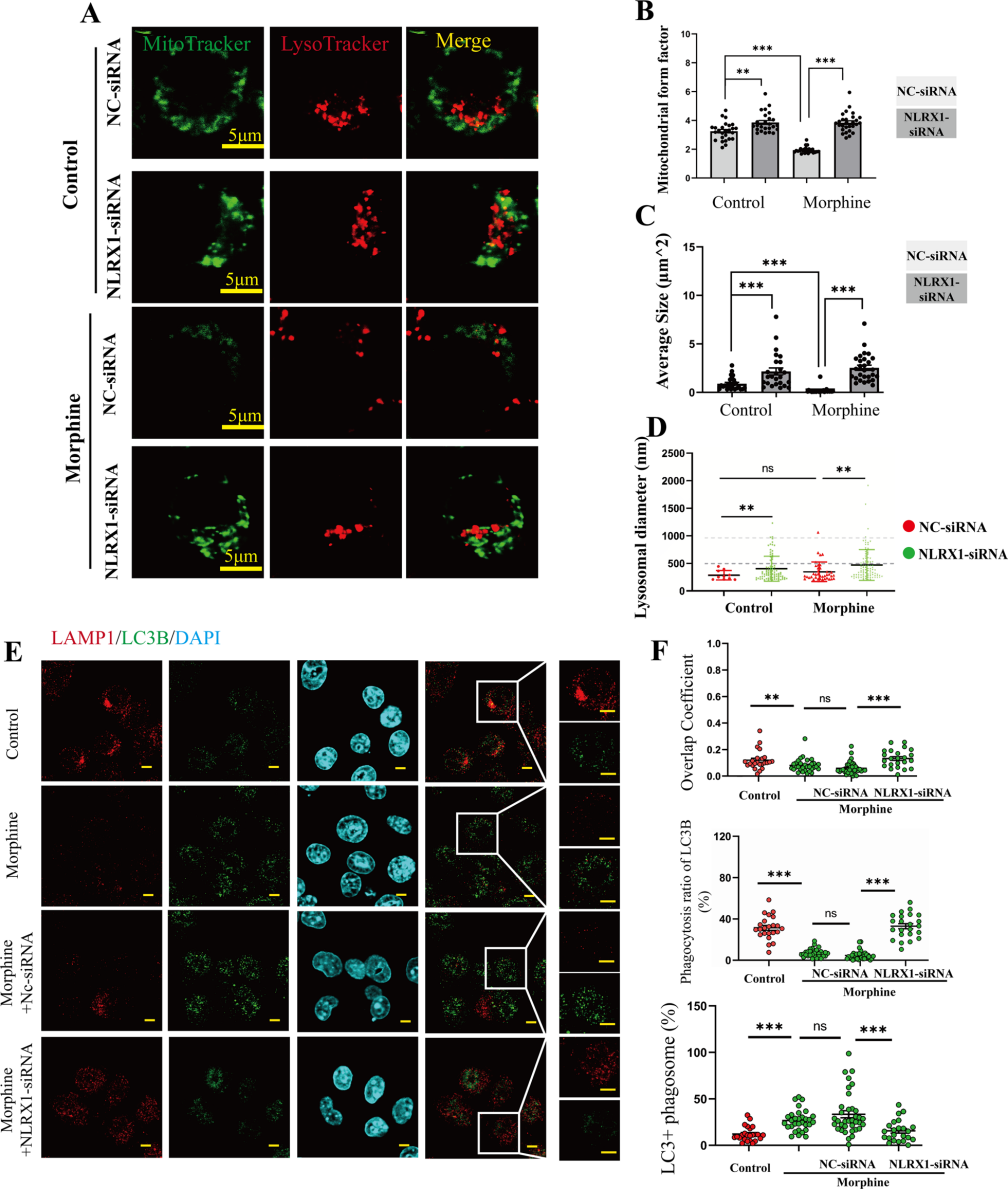
NLRX1-mediated incomplete mitophagy in morphine-treated BV2 cells. A Confocal microscopy analysis of MitoTracker Green Tm and LysoTracker Red DND-99 in BV2 cells of NC-siRNA or NLRX1-siRNA group at the absent or present of morphine. Bar = 5 μm. Quantitative graphs of mitochondrial form factors (**B**), average sizes (**C**) and lysosomal diameters (**D**) as measured by Image J (*n* > 25 cells). **E**–**F** Representative images of confocal microscopy analysis of LAMP1 and LC3B and quantitative analysis of overlap coefficients, lysosomal phagocytosis ratio of LC3B and LC3 + phagosomes (**F**, *n* = 24–36 cells). Bar = 2 μm. Data represent the mean ± SEM. Student's t-test or Mann–Whitney U test were used to measure significance between two groups. (* *p* < 0.05, ** *p* < 0.01, *** *p* < 0.001 and ns *p* > 0.05)

To further investigate the NLRX1-mediated mitophagy, MitoTracker Green Tm was used to stain mitochondria while LysoTracker Red DND-99 was used to stain lysosomes in BV2 cells (Fig. 3A). The mitochondrial morphologies were then analyzed by form factor (FF) and average size. FF = 1 represented a particle with a perfect circle and increased FF values were regarded as elongated mitochondria [19]. As expected, the mitochondrial FF values declined in morphine-treated BV2 cells (Fig. 3B), indicating fragmented mitochondria caused by morphine. Besides, upon pre-treatment with NLRX1-siRNA, the FF values increased in the absence or presence of morphine (Fig. 3B). Consistent with the findings, the average sizes of mitochondria decreased after morphine treatment while pre-treatment with NLRX1-siRNA rescued the decline (Fig. 3C). Overall, our results indicated that NLRX1 regulated mitochondrial fission to maintain homeostasis under normal conditions while morphine treatment aggravated NLRX1-mediated mitophagy.

Counterintuitively, the co-localization of mitochondria and lysosomes seemed to decline along with decreased amounts of lysosomes per cell after morphine treatment. The fusion of mitophagosomes with lysosomes followed by further proteolytic degradation was recognized as signature for the completion of mitophagy [7]. We then investigated whether NLRX1 manipulated complete mitophagy and facilitated elimination of fragmented mitochondria. The lysosomal diameters ranged from 0.2 to 2.5 μm as shown in Fig. 3D. It was accepted that the sizes of common lysosomes were no more than 1.0 μm, and we did not observe abnormal lysosomal vacuoles (> 3.0 μm) in morphine-treated cells [20]. Intriguingly, larger lysosomes were detected in those groups which were pre-treated with NLRX1-siRNA (Fig. 3D), thus we inferenced that NLRX1 played a role in lysosomal function. Therefore, we co-stained LAMP1 (lysosomal marker) and LC3B to further investigate mitochondrial–lysosomal crosstalk (Fig. 3E). Decreased LAMP1 intensity was observed in BV2 cells with morphine treatment. The overlap coefficients and lysosomal phagocytosis ratio of LC3B were decreased in morphine-treated BV2 cells, which were rescued by pre-treatment with NLRX1-siRNA (Fig. 3F). Then, the ratio of LC3^+^ phagosomes increased in the morphine group indicating remarkable accumulation of LC3B punctas, which was inhibited by NLRX1-siRNA. Together, these results indicated that NLRX1 might mediate morphine-induced dysfunctional mitochondrial–lysosomal crosstalk.

### Morphine induced NLRX1-mediated lysosomal dysfunction

**Fig. 4:**
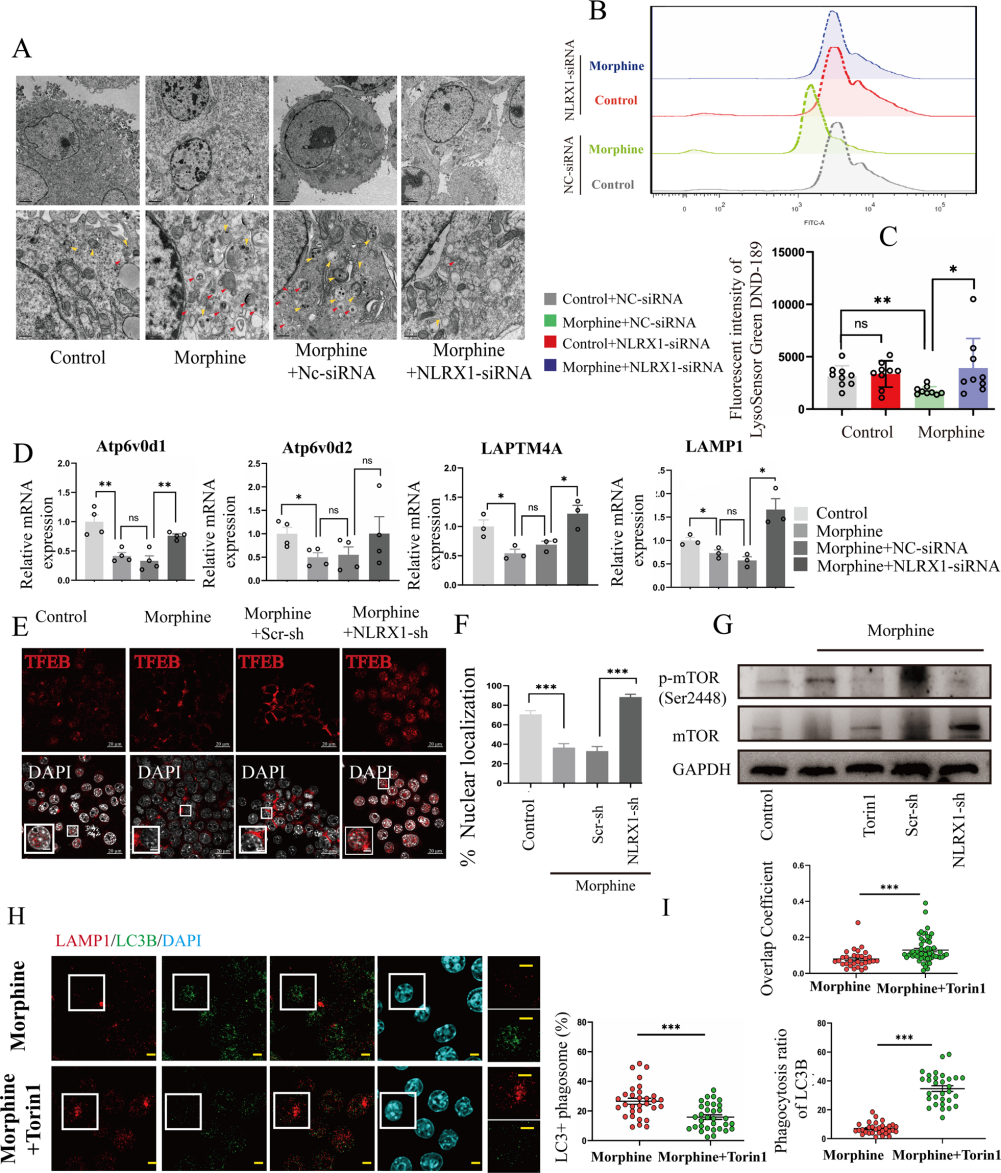
Torin1 treatment recovered the lysosomal function injured by morphine in BV2 cells. **A** The TEM analysis of ultrastructure of mitophagosomes (red arrowheads) and autolysosomes (yellow arrowheads). **B**, **C** The representative fluorescent intensity of LysoSensorTM Green DND189 (**B**) and quantitative analysis are shown in C as measured by flow cytometry (*n* = 9). **D** The mRNA expression of lysosome-related genes (*ATP6V0D1*, *ATP6V0D2, LAPTM4A* and *LAMP1*) as measured by qPCR analysis (*n* = 3–4). **E**, **F** The nuclear translocation of TFEB was measured by confocal microscopy analysis and the quantified results are shown in **F** (*n* = 25 random fields). **G **The activation of mTOR was measured by Western blots (*n* = 3). **H** Representative images of confocal microscopy analysis of LAMP1 and LC3B with or without Torin1 treatment in morphine-treated BV2 cells. Bar = 2 μm. **I** Quantitative analysis of overlap coefficients, lysosomal phagocytosis ratios of LC3B and LC3 + phagosomes in Fig. 3H (*n* > 30 cells). Data represent the mean ± SEM. Student's t-test or Mann–Whitney U test were used to measure significance between two groups (* *p* < 0.05, ** *p* < 0.01, *** *p* < 0.001 and ns *p* > 0.05)

To further manifest the issues, the morphologies of mitophagosomes and phagolysosomes were evaluated by electron microscopy assays (Fig. 4A). In the control group, most mitochondria were normal and mitophagosomes were seldom observed. In the morphine group and morphine + NC-siRNA group, increased mitochondria were encapsulated with double-membrane, indicating enhanced mitophagy activation and mitophagosome accumulation [21]. In addition, some phagolysosomes (mitophagosomes fused with lysosomes) were observed. In the morphine + NLRX1-siRNA group, the accumulation of mitophagosomes and phagolysosomes were tempered, supporting NLRX1-regulated mitophagy flux in morphine-treated BV2 cells. Thus, lysosomal PH indicator, LysoSensor™ Green DND189 was used to investigate the acidity of lysosomes. In contrast to the control group, the fluorescence intensity was decreased in the morphine group, indicating declined acidification of lysosomes (Fig. 4B, C). NLRX1-silencing restored the lysosomal acidity. We then investigated lysosomal function by monitoring lysosome-related genes. The mRNA levels which were detected included *ATP6V0D1* (facilitating lysosomal acidification), *ATP6V0D2* (facilitating mitophagosome–lysosome fusion) [22], *LAPTM4A* and *LAMP1* (facilitating lysosomal biogenesis) (Fig. 4D). After morphine treatment, the lysosome-related genes were all downregulated, suggesting morphine-induced lysosomal dysfunction. Pre-treatment with NLRX1-siRNA could reverse the downregulation of *ATP6V0D1*, *LAPTM4A*, and *LAMP1* without *ATP6V0D2*, suggesting NLRX1 might be dispensable for autophagosome–lysosome fusion. To understand the mechanisms of NLRX1-mediated lysosomal dysfunction, TFEB (transcription factor EB), a key regulator of lysosome biogenesis and activity [23, 24] was examined (Fig. 4E). Under normal condition, nuclear translocation of TFEB was commonly observed to maintain metabolic homeostasis. With morphine treatment, the nuclear translocation of TFEB was remarkably inhibited (Fig. 4F), indicating the inactivation of TFEB. NLRX1 silencing restored the nuclear translocation of TFEB, reflecting the interaction of NLRX1 and TFEB inactivation [19]. mTORC1 (mechanistic target of rapamycin kinase complex 1) phosphorylated the TFEB and prevented the nuclear translocation, thus leading to suppression of its catabolic function [25, 26]. We therefore wondered whether NLRX1 mediated the regulatory signaling of MTORC1 and TFEB in morphine-treated BV2 cells. Intriguingly, morphine boosted the mTORC1 activity (Fig. 4G, densitometric quantification in Additional file 2: Fig. S2E), accounting for the TFEB cytoplasmic accumulation. Torin1, a potent mTORC1 inhibitor, was utilized to suppressed the phosphorylation of mTOR. Similarly, the mTORC1 activity was hampered by NLRX1 silencing. Our data indicated NLRX1 might regulate the mTORC1 activity, participating in a lysosomal dysfunction mechanism by blocking the activation of TFEB.

### Restoring lysosomal function contributed to complete mitophagy in morphine-treated microglia

Subsequently, Torin1 was then utilized to restore lysosomal function (Additional file 3: Fig. S3A). As a result, the downregulations of lysosomal genes were significantly recovered by Torin1 for 15 min. We applied the treatment to morphine-treated BV2 cells and performed co-staining of LC3B and LAMP1 for co-localization analysis (Fig. 4H). Compared to morphine treatment alone, the addition of Torin1 significantly inhibited the formation of LC3B punctas and enhanced the intensity of LAMP1 fluorescence in BV2 cells. Besides, the overlap coefficients, and lysosomal phagocytosis ratio of LC3B were enhanced by Torin1 while the ratio of LC3^+^ phagosomes was decreased (Fig. 4I). Taken together, we could conclude that morphine induced insufficient mitophagy owing to lysosomal dysfunction, and NLRX1 participated in the crosstalk between mitochondria and lysosomes by interference with the generation and acidification of lysosomes. Consequently, NLRX1 mediated incomplete mitophagy in morphine-treated BV2 cells, with a concomitant accumulation of mitophagosomes and immature autolysosomes.

### NLRX1-mediated incomplete mitophagy facilitated microglial immunosuppression after morphine treatment

**Fig. 5:**
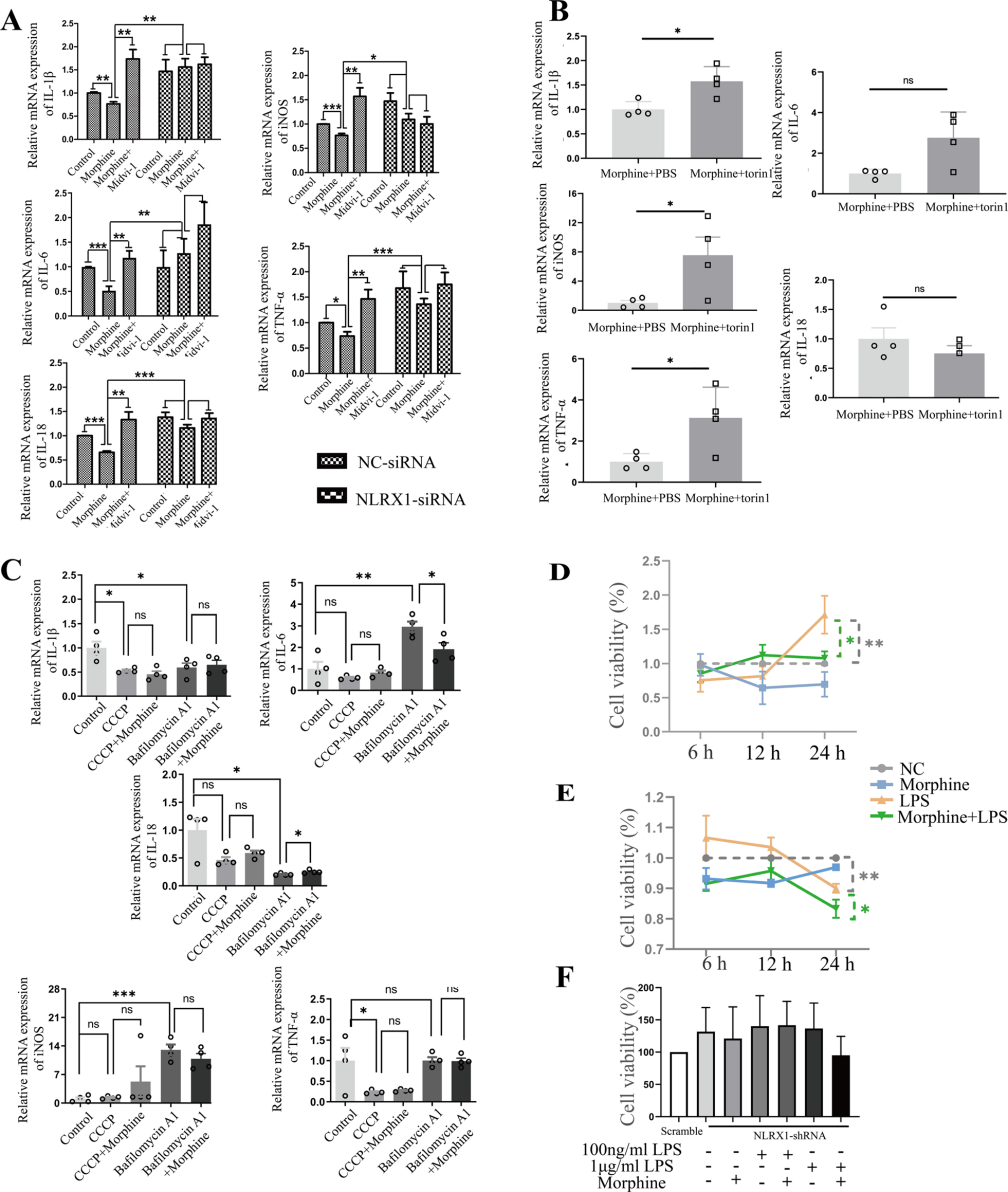
NLRX1-mediated incomplete mitophagy facilitated morphine induced microglial immunosuppression and susceptibility to infection. **A** NLRX1 regulated the mitophagy-induced immunosuppression in morphine-treated BV2 cells (*n* = 4–8), as measured by qPCR analysis. **B** Torin1 treatment partly rescued the inhibition of proinflammatory cytokines including *IL-1β*, *TNF-α* and *iNOS* (*n* = 4). **C** After treatment with CCCP or Bafilomycin A1 at the absent or present of morphine, the expressions of proinflammatory cytokines were measured by qPCR analysis (*n* = 4). **D**, **E** The cell viability was detected in BV2 cells with 100 ng/ml LPS treatment (**D**, *n* = 3) or 1ug/ml LPS treatment (**E**, *n* = 3). **F** LPS challenge was performed in scrambled-shRNA or NLRX1-shRNA primary microglial cells. The cell viability was measured by CCK8 (*n* = 3). Data represent the mean ± SEM. Student's t-test or Mann–Whitney U test were used to measure significance between two groups (* *p* < 0.05, ** *p* < 0.01, *** *p* < 0.001 and ns *p* > 0.05)

To confirm immunosuppression in microglia caused by morphine, the expression of proinflammatory cytokines was detected by qPCR assay. Markedly, after morphine treatment, the mRNA expression of *IL-1β*, *IL-6*, *IL-18*, *TNF-α*, and *iNOS* were all significantly downregulated in BV2 cells, which were rescued by pre-treatment with NLRX1-siRNA (Fig. 5A), indicating that NLRX1 participated in morphine-induced immunosuppression in microglia. Then, Mdivi-1, a mitochondrial fission inhibitor, was utilized as a mitophagy inhibitor [27]. With deficiency of mitophagy caused by Mdivi-1, the proinflammatory cytokines *IL-1β*, *IL-6*, *IL-18*, *TNF-α*, and *iNOS* were all significantly upregulated in morphine-treated BV2 cells (Fig. 5A). Additionally, pre-treatment with NLRX1 siRNA yielded no significant observable difference between the morphine group and morphine + Mdivi-1 group. The data supported the viewpoint that NLRX1-mediated mitophagy contributed to immunosuppression in microglia caused by morphine. Further, the downregulations of *IL-1β*, *TNF-α*, and *iNOS* caused by morphine were reversed by Torin1, supporting the importance of lysosomal function in the maintenance of normal immunity which could be impaired by morphine (Fig. 5B) [28]. Thus, our data revealed that NLRX1-mediated incomplete mitophagy might account for the immunosuppression caused by morphine in microglia.

Then, we investigate whether CCCP-induced complete mitophagy led to microglial immunosuppression (Fig. 5C). Virtually, no differences were observed between the CCCP group and control group except *IL-1β* and *TNF-α*, which were more inclined to be regulated by elimination of mitochondria [29]. Bafilomycin A1, a selective vacuolar-type ATPase inhibitor, was reported to inhibit lysosomal acidification and fusion [30]. With the treatment of Bafilomycin A1, the mRNA expressions of *IL-6* and *iNOS* were upregulated, with a concomitant decrease of *IL-1β* and *IL-18*. In addition, remarkable downregulation of *IL-6* and upregulation of *IL-18* was detected in the morphine + bafilomycin A1 group, in contrast to the bafilomycin A1 group (Fig. 5C). Presumably, bafilomycin A1 was more efficacious than morphine in inhibiting v-ATPase, thus leading to complicated and elusive inflammatory response. Also, previous studies reported that bafilomycin A1 did not lead to the accumulation of ROS (reactive oxygen species), but disrupted the degradation of ubiquitinated NLRP3 inflammasome, which might account for the downregulation of *IL-1β* and* IL-18* [31, 32]. Taken together, NLRX1-mediated incomplete mitophagy in microglia accounted for more integral immunosuppression, compared to CCCP-induced complete mitophagy and bafilomycin A1-induced lysosomal dysfunction.

### NLRX1-mediated immunosuppression accounted for the susceptibility to infection

Then, lipopolysaccharide (LPS) challenge assay was performed in primary microglial cells. 100 ng/mL LPS led to microglial proliferation (Fig. 5D) while 1 ug/mL LPS (Fig. 5E) decreased cell viability in a time-dependent manner, peaking at 24 h. Subsequently, cells were, respectively, treated with morphine and LPS for 24 h. Virtually, the viability of microglial cells which received morphine treatment remained similar to the control. However, dampened viability was observed in the LPS + morphine group, compared to 100 ng/mL LPS or 1 ug/mL LPS (Fig. 5D, E). Then, optimal short hairpin RNAs (shRNAs) targeting NLRX1 on primary microglia was chosen according to qPCR analysis (Additional file 1: Fig. S1D). NLRX1-silenced microglial cells tended to be resistant to LPS stimulation. No significant difference was observed between the LPS group and LPS + morphine group (Fig. 5F). Taken together, NLRX1-mediated microglial immunosuppression might contribute to susceptibility to infection.

### Chronic morphine treatment disturbed host systemic immunity in mice

**Fig. 6:**
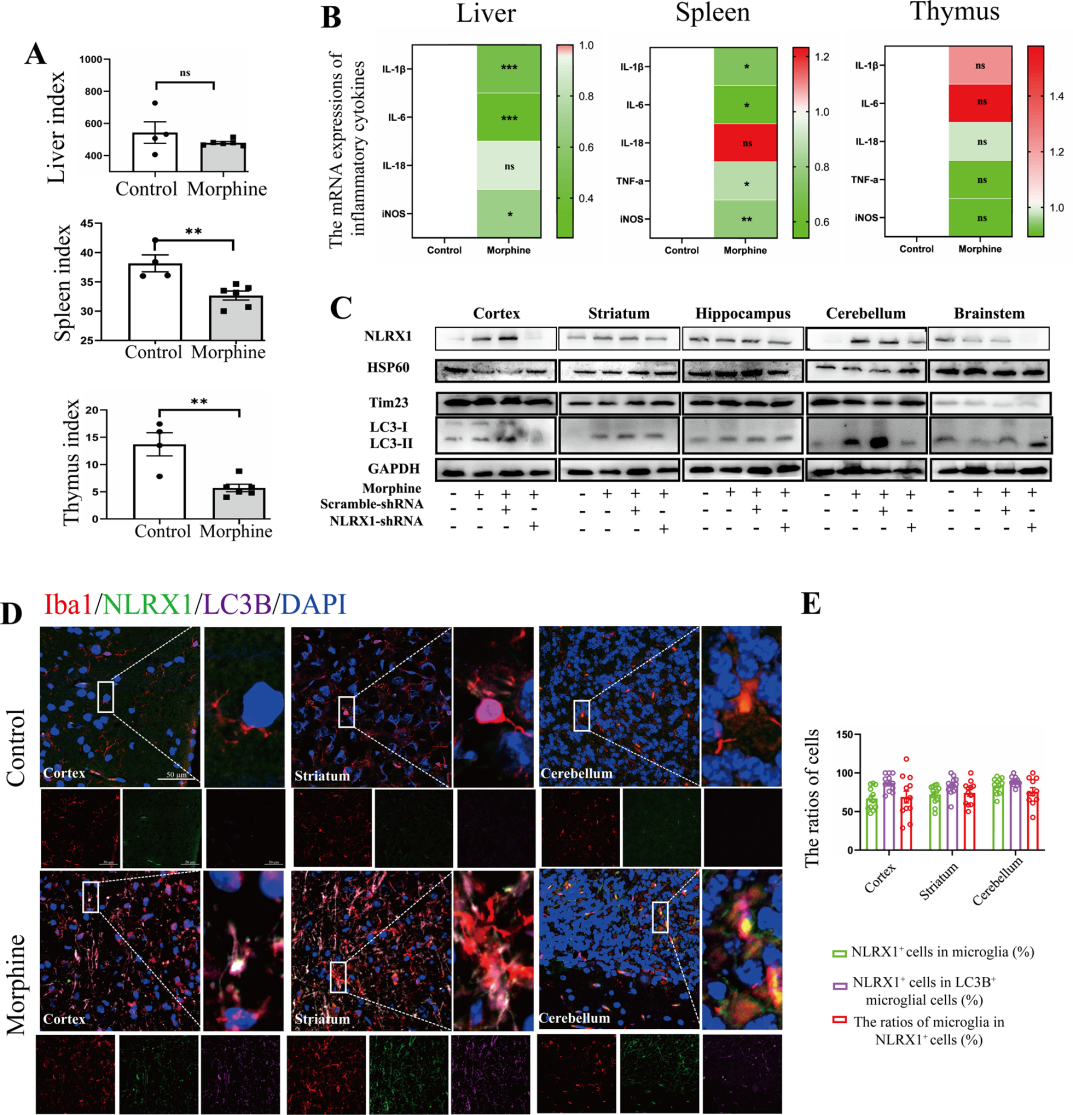
Chronic morphine disturbed host systemic immunity and induced microglial mitophagy in mice. **A** The quantitative analysis of organ indexes in mice (*n* =46). **B** The heat maps of liver (left), spleen (middle) and thymus (right) indicated the mean values of mRNA expression of inflammatory cytokine genes (*n* = 4–6). **C** Representative Western blots of Hsp60, Tim23 and LC3 in cortex, striatum, hippocampus, cerebellum and brainstem in control or morphine-treated mice (*n* = 3–4). **D** Confocal microscopy analysis of Iba1 (red), NLRX1 (green), LC3B (magenta) and DAPI (blue) in cortex, striatum and cerebellum in control or morphine-treated mice. Bar = 50 μm. **E** The ratios of NLRX1^+^ cells in microglia, NLRX1^+^ cells in LC3B + microglial cells and microglia in NLRX1 + cells in **D** were analyzed (*n* = 11–12 fields). Data represent the mean ± SEM. Two-sided Student’s t tests were used to measure significance between two groups (* *p* < 0.05, ** *p* < 0.01, *** *p* < 0.001 and ns *p* > 0.05)

To delineate the host immunity in morphine-treated mice, liver index, spleen index, and thymus index were measured. In accord with previous research, chronic morphine treatment led to the atrophy of immune organs (Fig. 6A), underlying the impaired host immune defense system against pathogens [16]. No detectable change was observed in liver index in the morphine group. Subsequently, mRNA expressions of inflammatory cytokines were analyzed in the liver (Fig. 6B, left), spleen (Fig. 6B, middle), and thymus (Fig. 6B, right). With morphine treatment, *IL-1β*, *IL-6*, and *iNOS* were all downregulated in the liver and spleen (Fig. 6B), while they remained unchanged in the thymus.

### Chronic morphine treatment induced microglial mitophagy in cortex, striatum, and cerebellum

**Fig. 7:**
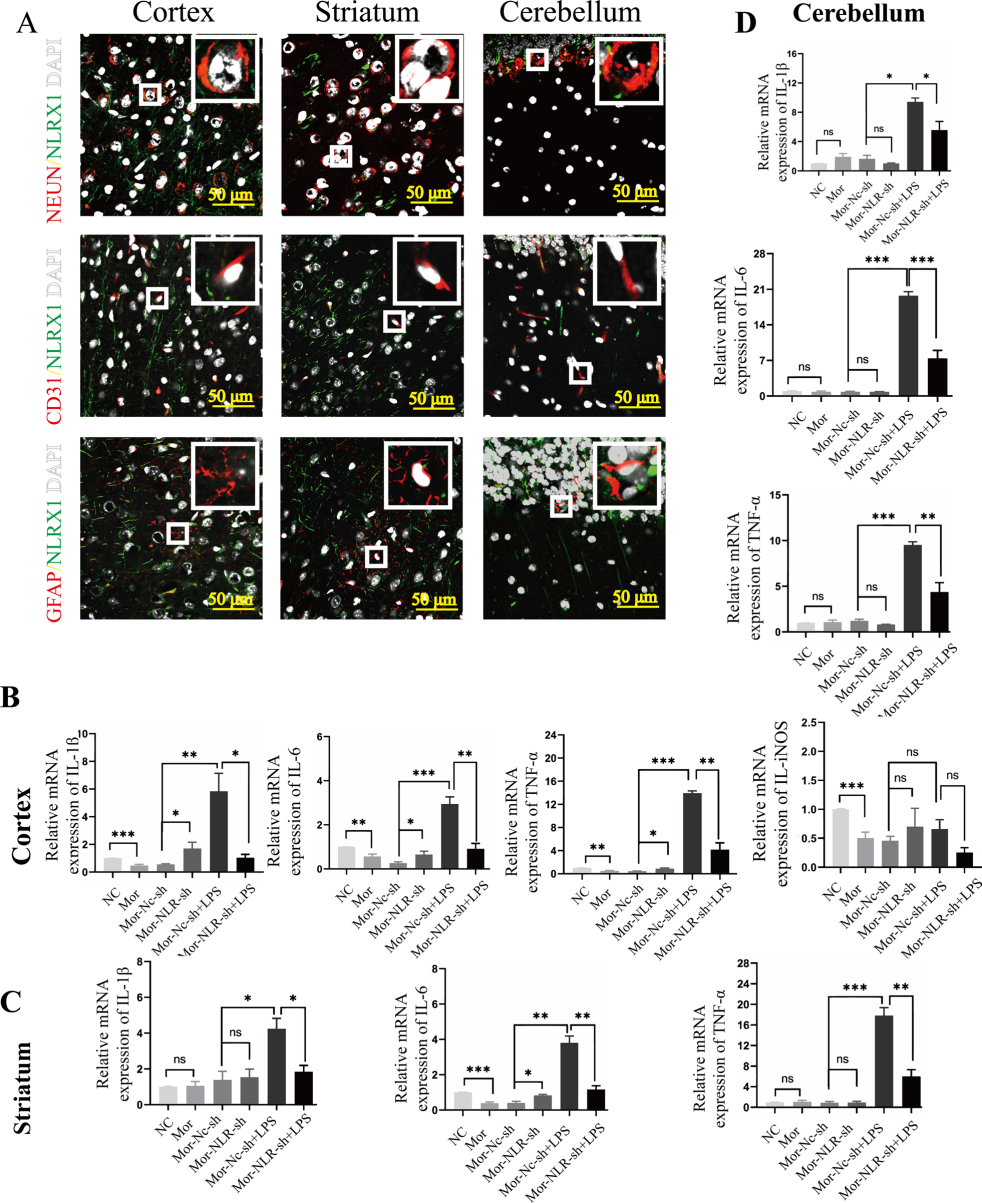
Chronic morphine induced NLRX1-mediated immunosuppression in brain. **A** Confocal microscopy analysis of NLRX1 (green) and NeuN (red), CD31 (red) or GFAP (red). Bar = 50 μm. **B**, **C**, **D** The mRNA expression of inflammatory cytokine genes were analyzed in cortex (**B**), striatum (**C**) and cerebellum (**D**) in mice (*n* = 3–5). Quantitative graphs are shown by mean ± SEM. Data represent the mean ± SEM. Student's t-test or Mann–Whitney U test were used to measure significance between two groups (* *p* < 0.05, ** *p* < 0.01, *** *p* < 0.001 and ns *p* > 0.05)

To confirm NLRX1-mediated mitophagy in vivo, lentivirus of scrambled or NLRX1-shRNA were generated and injected intracerebroventricularly into male C57BL/6 mice. The protein level of NLRX1 increased remarkably in the cortex, striatum, and cerebellum in the morphine group (Fig. 6C, densitometric quantification data in Additional file 3: Fig. S3A). NLRX1-shRNA visibly inhibited the expression of NLRX1 in the cortex, striatum, cerebellum, and brainstem, confirming the efficiency of NLRX1-shRNA lentivirus in morphine-treated mice. Then, the protein levels of HSP60 and Tim23 were both decreased in the cortex, striatum, and cerebellum after chronic morphine exposure, which was rescued by NLRX1 silencing (Fig. 6C). Next, we aimed to find out the brain regions and cell types in which NLRX1-mediated mitophagy mainly occurred in mice. Fluorescent staining showed that the protein expressions of NLRX1 and LC3B were both increased in the cortex, striatum, and cerebellum, consistent with the results of western blotting assay (Fig. 6D, the images of hippocampus and brainstem are shown in Additional file 3: Fig. S3C). Although the expression of LC3B and conversion of LC3 I to II was increased in the hippocampus, NLRX1 and mitochondrial protein remained unchanged, implying autophagy, rather than mitophagy, should account for the upregulation of LC3B [33]. Furthermore, the co-localization of NLRX1 and LC3B was mainly observed in microglial cells in the above-mentioned brain regions (Fig. 6E), accounting for major cell population of NLRX1 + or NLRX1 + LC3B + cells. To further confirm the results, we then co-stained NLRX1 with NeuN, CD31 or GFAP in the cortex, striatum, and cerebellum (Fig. 7A). As expected, minimal co-localization was observed in the above-mentioned brain regions, providing evidence that NLRX1-mediated mitophagy in murine brains mainly occurred in microglial cells.

### NLRX1-mediated microglial immunosuppression led to exaggerated sepsis in brain

To substantiate the cerebral immunosuppression mediated by NLRX1, proinflammatory cytokines were analyzed in the abovementioned brain regions (Fig. 7B–D, data without significant difference are shown in Additional file 3: Fig. S3D). Remarkably, *IL-1β*, *IL-6*, *TNF-α*, and *iNOS* in the cortex and *IL-6* in the striatum of morphine-treated mice were downregulated and NLRX1-silencing could rescue the downregulations of *IL-1β*, *IL-6*, *and TNF-α*. LPS challenge led to exaggerated inflammation including upregulations of *IL-1β, IL-6*, *and TNF-α*, which were significantly ameliorated by NLRX1-silencing (Fig. 7B–D). Excess accumulation of the above mentioned proinflammatory cytokines could exert inflammatory neuronal injury [34, 35]. These data clearly supported that NLRX1 contributed to the cerebral susceptibility of morphine-treated mice to infection.

## Discussion

Morphine-induced immunosuppression has been widely discussed in the past, but the underlying mechanisms remained unclear yet [16]. In addition to the peripheral immune system, the brain-resident system is also an important susceptible region to pathogen infection after chronic morphine exposure which will cause poor prognosis for patients [36]. In this study, we demonstrated that NLRX1-mediated insufficient mitophagy facilitated microglial immunosuppression after morphine treatment, which might be responsible for the fragility to invading pathogens.

Firstly, enhanced NLRX1 expression and mitophagy activation were concomitantly observed in microglia treated with morphine for 24 h. Furthermore, because a distinctive LIR motif for LC3 binding was a prerequisite for receptor-mediated mitophagy, we demonstrated the binding of NLRX1 to LC3, serving as a mitophagy receptor. NLRX1-silencing rescued the morphine-induced mitophagy in microglia. Presumably, neither NLRX1 nor mitophagy seemed to function in astrocytes after morphine treatment, which indicated that microglia might be the main inflammatory cell type for morphine-induced mitophagy in the CNS. It was not surprising that there were cell type-specific differences after morphine treatment. It has been demonstrated that astrocytes were resistant to 1 μM morphine induced cytotoxicity but not microglia [37, 38]. More importantly, morphine tended to protect astrocytes from glutamate-induced apoptosis [39] and activated the astrocytic μ receptor but not microglial to promote the release of CCL5, which exhibited a neuroprotective property during HIV infection [40]. Therefore, morphine mediates multiple effects in different cells in CNS. More importantly, there was limited evidence demonstrated the direct role of NLRX1 in the astrocytic inflammatory response. It has been substantiated neurotoxic astrocytes were subsequently induced by activated microglia [41]. Thus, an alternative explanation assumed that NLRX1 might inhibit microglial activation, resulting in interrupting the generation of neurotoxic astrocytes [42]. However, future work is required to address whether NLRX1 could directly regulate the astrocytic inflammatory response. The *Δψm* remained unchanged and thus the PINK1–Parkin axis might not be responsible for morphine-induced mitophagy, counterintuitively. No detectable change of NLRX1 expression was observed in CCCP-treated BV2 cells. These results indicated that NLRX1-mediated mitophagy was activated by morphine in a specific manner, independently of the PINK1–Parkin pathway.

Secondary, we focused on the mitophagic flux regulated by NLRX1 in morphine-treated BV2 cells. Our results showed that morphine disturbed lysosomal function, including lysosomal acidification, mitophagosome–lysosome fusion, and lysosomal biogenesis. LC3B punctas accumulated in the cytosol which resulted from enhanced LC3-decorated mitophagosomes and insufficient lysosomal degradation. NLRX1-silencing alleviated the lysosomal dysfunction through promotion of lysosomal generation and acidification, but failed to entirely restore the mitophagosome–lysosome fusion. These results implied the NLRX1-mediated mitophagy might be incomplete with lysosomal dysfunction. TFEB is a master modulator of lysosomal catabolic function [43]. TFEB regulated the lysosomal activity via binding to conserved coordinated lysosomal expression and regulation (CLEAR) motif of targeted genes [23]. Our results suggested that NLRX1 might suppress the nuclear translocation of TFEB. The association of NLRX1 deficiency and activation of TFEB has also been demonstrated by a previous study [19]. However, detailed mechanisms remained unknown. mTORC1 has a critical role in suppressing the lysosomal function by inactivating TFEB [44]. Herein, we supposed the TFEB subcellular localization regulated by NLRX1 might via the mTOR pathway. Our results confirmed our hypotheses and the phosphorylation of mTOR was enhanced in morphine-treated microglia, which could be inhibited by NLRX1 silencing. Recent evidence has demonstrated the association of NLRX1 and mTORC1 activity in lung aging [45]. Our data shed light into the notion that NLRX1 regulated the lysosomal function might via mTORC1-TFEB signaling. However, further work is required to further demonstrate the interaction of NLRX1 and mTORC1 function.

It has been reported that lysosomal function was essential for maintaining normal innate immunity and pathogen resistance [46]. The complex interaction between mitochondria and lysosomes is essential for homeostasis of the immune system [19]. Consistently, we observed the downregulation of inflammatory cytokines (*IL-β*, *IL-6*, *IL-18*, *TNF-α*, and *iNOS*) in morphine-treated BV2 cells, which indicated immunosuppression after chronic morphine exposure, and this could be rescued by NLRX1-silencing. Hence, this phenomenon supported the viewpoint that NLRX1-mediated incomplete mitophagy led to immunosuppression in morphine-treated microglia. Mdivi-1 was then used as a mitophagy inhibitor to confirm the inflammatory inhibition of mitophagy following morphine treatment. As expected, deficiency of mitophagy significantly increased the expressions of inflammatory cytokines *IL-1β*, *IL-6*, *IL-18*, *TNF-α*, and *iNOS* in the presence of morphine, while NLRX1-silencing invalidated the effect of Mdivi-1. Therefore, it was confirmed that morphine induced immunosuppression through NLRX1-mediated mitophagy.

Since NLRX1-mediated mitophagy caused by morphine was incomplete, we then aimed to determine whether correcting the lysosomal dysfunction to repair mitophagy could rescue immunosuppression in microglia. As excepted, Torin1 contributed to partial upregulation of inflammatory cytokines (*IL-1β*, *TNF-α*, and *iNOS*), supporting our hypothesis. Unlike CCCP-induced completed mitophagy or bafilomycin A1-induced lysosomal dysfunction alone, morphine was responsible for the extensive inhibition of proinflammatory cytokines and susceptibility to infections in microglia. The coordination of mitochondrial ligands and innate immune sensors, such as TLRs and cGAS/STING, mediated host immune responses to pathogen-associated molecular patterns (PAMPs) or damage-associated molecular patterns (DAMPs). TLRs signaling pathway has been reported to trigger secretion of inflammatory cytokines, such as *IL-1β*, *IL-6*, and *TNF-α*. The immunogenic capabilities of damaged mitochondria have also been underscored, resulting in excessive mtROS production and accumulated cytosolic mtDNA. NLRP3 inflammasome was subsequently activated and induced accumulation of *IL-1β* and *IL-18* [47]. Inhibiting mitophagy initiation improved immune defense against viruses by enhanced activation of the NLRP3 inflammasome [48]. Additionally, multiple inflammatory cytokines might serve as downstream factors of *IL-1β*, such as *TNF-α*, *IL-6*, and *iNOS* [49]. Therefore, mitochondria served as platforms of manipulation in integrating complex signals to trigger immune activation [50]. Lysosomes degraded cytoplasmic constituents including defective mitochondrion for recycling, reconstitution and modification. Insufficient lysosomal function interrupted the mitophagic flux and broke intrinsic immune homeostasis. Additionally, lysosomal maturation served as a critical role in the elimination of invading pathogens and incomplete mitophagy promote intracellular pathogen infection [51]. Ultimately, incomplete mitophagy potentiated the immune deficiency. Therefore, morphine-treated microglial cells should be more vulnerable to pathogenic challenge such as bacterial LPS. However, the detailed mechanisms underlying how NLRX1-mediated mitophagy facilitated downregulation of inflammatory cytokines required further elucidation.

Thirdly, we successfully generated a mouse model for chronic morphine induced immunosuppression. The suppressed systemic immunity caused by morphine group was observed in peripheral organs by the decline of the spleen index and thymus index. Besides, the inhibition of inflammatory cytokines (IL-1β, IL-6, and iNOS) in the liver and spleen induced by morphine might account for the vulnerability to infections. According to the NLRX1 expression in the ‘HUMAN PROTEIN ATLAS’ (https://www.proteinatlas.org/ENSG00000160703-NLRX1/brain), we detected the NLRX1 expression and mitophagy in the cortex, striatum, hippocampus, cerebellum, and brainstem to confirm the cellular findings in vivo. We demonstrated that NLRX1-mediated mitophagy was, respectively, enhanced in the cortex, striatum, and cerebellum, after exposure to chronic morphine stimulation. No significant difference was observed in the hippocampus and brainstem between the control and morphine group. Microglial cells, rather than neuron, astroglia or vascular endothelial cells, were then characterized as the main cell type, where NLRX1-mediated mitophagy occurred in brain of morphine-treated mice. Our findings were consistent with previous reports that NLRX1 acted as an enigmatic regulator in immune cells [52].

Moderate proinflammatory response after infection helps to clean the invading pathogen and therefore transition of M1 microglia to M2 phenotype helps to repair the damage [53, 54]. Morphine exposure has been demonstrated to disturb the microglial secretion of inflammatory cytokines, such as *TNF-α*, *IL-6*, and *CCL2/MCP-1* [55]. The pathological or diseased microglia might lead to the aggravated inflammatory storm or constant presence of inflammation in response to infection [45, 56, 57]. The immunological exhaustion accounts for the vulnerability of infection and deteriorated inflammatory damage in brain. In addition, microglia in different brain regions displayed diverse characteristics under pathogenic challenge. They have been demonstrated to function as immunoregulatory mediators in the cortex, striatum, and cerebellum [58, 59]. Herein, it was plausible that NLRX1 functioned as a negative immune regulator and aggravated septic injury in brain of chronic morphine-treated mice, mainly in the cortex, striatum, and cerebellum. There are some issues remained to address. A recent report demonstrated that the brain could modulate adaptive immunity responses directly in immune organs [60]. In addition, mounting evidence indicate that damage in CNS might contribute to impaired immune system and thus facilitates immunodepression, increasing the risk of infections [61]. We therefore speculated a possible feedback loops between the brain and the immune system in the mouse after morphine treatment. In addition, whether and how NLRX1-mediated immunodepression in microglia in brain plays a role in peripheral immune system remains unknown.

## Conclusion

**Fig. 8:**
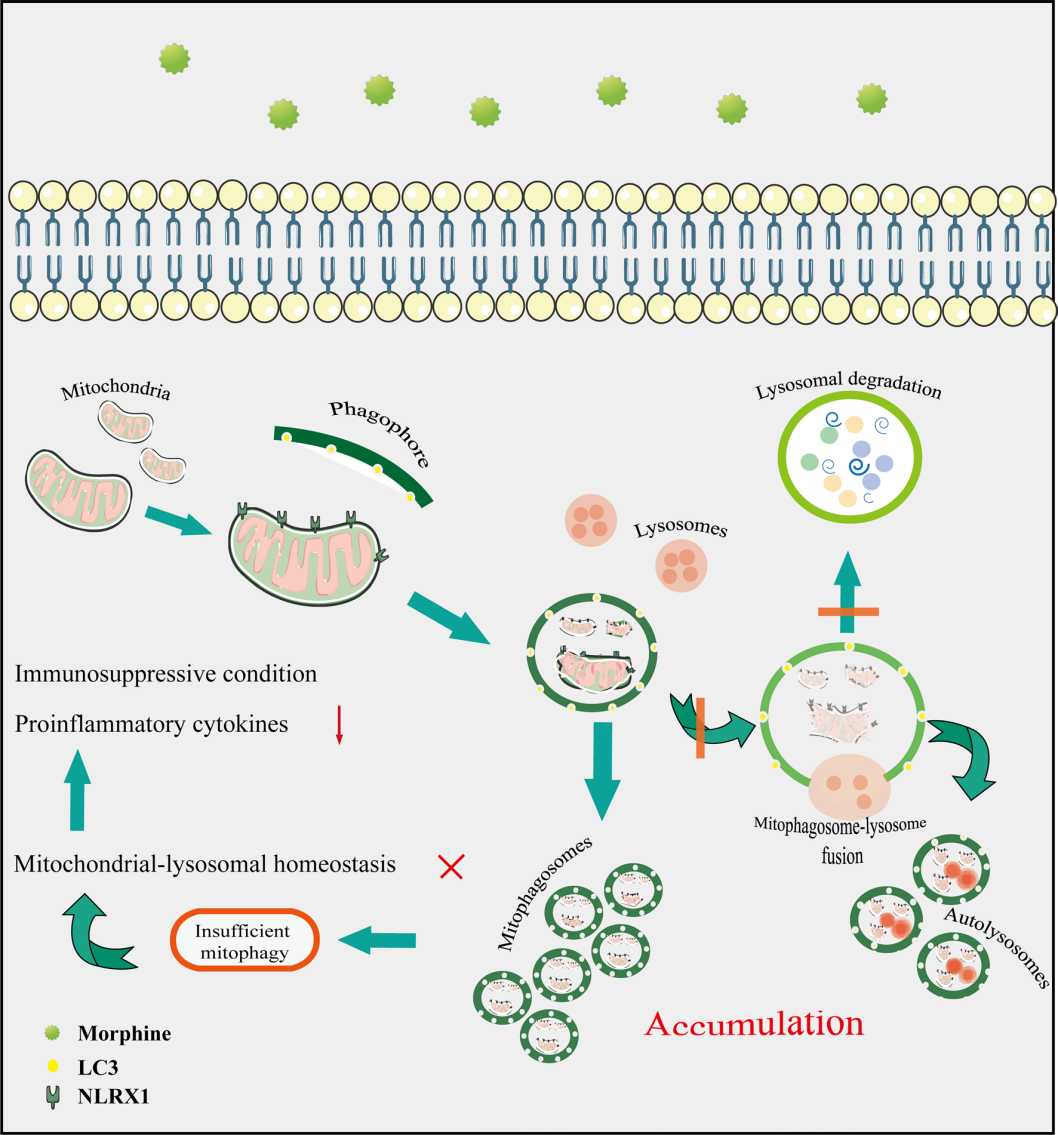
Schematic model of NLRX1-mediated insufficient mitophagy in morphine-treated microglial cells. Morphine enhanced the ability of NLRX1-mediated mitophagy in a LC3-dependent manner, while impairing lysosomal function including generation, mitophagosome–lysosome fusion and acidification, thus leading to disturbance of mitochondrial–lysosomal homeostasis. NLRX1-mediated incomplete mitophagy in morphine-treated microglial cells ultimately contributed to microglial immunosuppressive condition

In summary, we delineated the NLRX1-mediated incomplete mitophagy in morphine-treated microglial cells, which facilitated the immunosuppression and vulnerability for pathogenic challenge (Fig. 8). In chronic morphine-treated mice, NLRX1 might manipulate mitophagy in microglial cells of specific brain regions, including the cortex, striatum, and cerebellum where NLRX1 mediated inflammatory response to pathogenic challenges such as LPS. Overall, our study substantiated a new concept that NLRX1-mediated mitophagy in microglial cells contributed to morphine-induced immunosuppression in the brain. However, further elucidations are still required.

## Supplementary Information

**Additional file 1: Figure S1.** The extended data in primary microglia and astrocyte. (A-B) The purity of primary microglia (A, Iba1, red) and primary astrocytes (B, GFAP, red) were both > 95% (*n* = 12 random fields). Bar = 160 μm. (C) The quantitative graphs of Fig. 2B (*n* = 3) (D) Lentiviral short hairpin RNA (shRNA)-mediated knockdown of NLRX1 was used in primary microglia (*n* = 4) and optimal shRNAs targeting NLRX1 on primary microglia was chosen according to qPCR analysis and sequence c was chosen. Quantitative graphs were shown by mean ± SEM. Two-sided Student’s t tests were used to measure significance between two groups. (*p < 0.05, **p < 0.01, ***p < 0.001 and ns *p* > 0.05).

**Additional file 2: Figure S2.** The quantitative graphs of Western blots of mitophagy in BV2 and MA cells. (A) represented the quantitative graphs of Fig. 1D (*n* = 3–6), I (*n* = 6) and (B) represented Fig. 1J (*n* = 5). (C) represented the quantitative graphs of Fig. 1K (*n* = 3). (D) represented the quantitative graphs of Fig. 2D (*n* = 3). (E) represented the quantitative graphs of Fig. 4G (*n* = 3). Quantitative graphs were shown by mean ± SEM. Student's t-test or Mann–Whitney U test were used to measure significance between two groups. (* *p* < 0.05, ** *p* < 0.01, *** *p* < 0.001 and ns *p* > 0.05).

**Additional file 3: Figure S3.** Supplementary data for Figures. (A) Optimal time for Torin1 treatment including 15 min, 30 min and 1 h was chosen as measured by qPCR analysis of lysosome-related genes (*n* = 3–4). (B) represented the quantitative graphs of Fig. 6C (*n* = 3–4). (C) Confocal microscopy analysis of Iba1 (Red), NLRX1 (Green), LC3B (Magenta) and DAPI (Blue) in hippocampus and brainstem in control or morphine-treated mice. Bar = 50 μm. (D) The mRNA expression of *IL-18* and *iNOS* were analyzed in cortex (up), striatum (left) and cerebellum (right) in mice (*n* = 3–5). Quantitative graphs were shown by mean ± SEM. Student's t-test or Mann–Whitney U test were used to measure significance between two groups. (* *p* < 0.05, ** *p* < 0.01, *** *p* < 0.001 and ns *p* > 0.05).

**Additional file 4: Figure S4.** The whole blots used for Western blot analysis in the article and the blots used in the figures were marked in red frame.

## Data Availability

The datasets used and/or analyzed during the current study are available from the corresponding author on reasonable request.

## Abbreviations

NLRX1: NOD-like receptor X1
LC3: Microtubule-associated proteins light chain 3
LIR: LC3 interacting region
LPS: Lipopolysaccharide
CCCP: Carbonyl cyanide m-chlorophenylhydrazone
mtDNA: Mitochondrial DNA
IP: Immunoprecipitation
*Δψm*: Mitochondrial membrane potential
FF: Form factor
LAMP1: Lysosomal associated membrane protein 1
ROS: Reactive oxygen species
